# Assessing the Anxiolytic and Relaxation Effects of *Cinnamomum camphora* Essential Oil in University Students: A Comparative Study of EEG, Physiological Measures, and Psychological Responses

**DOI:** 10.3389/fpsyg.2024.1423870

**Published:** 2024-07-26

**Authors:** Xiangfei Gong, Yujun Yang, Tong Xu, Dongsheng Yao, Shengyu Lin, Weiyin Chang

**Affiliations:** ^1^College of Forestry, Fujian Agriculture and Forestry University, Fuzhou, China; ^2^Laboratory of Virtual Teaching and Research on Forest Therapy Specialty of Taiwan Strait, Fujian Agriculture and Forestry University, Fuzhou, China

**Keywords:** *Cinnamomum camphora*, essential oil, aromatherapy, electroencephalogram, natural therapy

## Abstract

**Background:**

*Cinnamomum camphora* is a commercially important tree species in China, and it’s also a common native tree in the forests of southern China. However, literature on the impact of *Cinnamomum camphora* essential oil (CCEO) on human psychophysiological activity is scarce. Hence, the primary objective of this study was to examine the effect of exposure to CCEO on the functioning of the human autonomic nervous system, electroencephalographic (EEG) activity, and emotional state.

**Methods:**

Forty-three healthy university students participated. The data collected included heart rate (HR), blood pressure (BP), pulse rate, blood oxygen saturation (SpO_2_), electroencephalographic (EEG) activity, and the results of the Profile of Mood States (POMS) test.

**Results:**

A drop in diastolic pressure (DBP) and pulse rate was also noticed after participants inhaled CCEO. Furthermore, EEG studies have demonstrated notable reductions in absolute beta (AB), absolute gamma (AG), absolute high beta (AHB), and relative gamma (RG) power spectra during exposure to CCEO. Conversely, the relative theta (RT) and power spectra values showed a significant increase. Additionally, the finding from POMS indicated that the fragrance evoked positive emotions and suppressed negative feelings.

**Conclusion:**

The results suggest that exposure to CCEO may promote mental and physical relaxation, facilitate cognitive processes such as memory and attention, and enhance mood states.

## 1 Introduction

Essential oils (EOs) are traditionally secondary metabolites extracted from various natural plant tissues that still retain the distinctive characteristics of the raw material (Yu et al., 2022). They are complex mixed compounds with alcohols, aldehydes, ketones, ethers, esters, acids, and oxides (Kim et al., 2018). Aroma-therapy is a form of therapy that focuses on the psychological impacts of EO scents and the physiological effects of inhaling its volatile chemicals (Lin et al., 2022). Since the ancient era, EOs have been utilized to promote humans’ physiological and psychological well-being (Angelucci et al., 2014). EOs and fragrance compounds have been employed in traditional medicine, aromatherapy, and herbal medicine to address a range of psychological and physical ailments, including but not limited to headaches, pain, insomnia, eczema, stress-induced anxiety, depression, and digestive issues (Hedayat and Tsifansky, 2008; Sowndhararajan and Kim, 2016).

Electroencephalography (EEG) is widely used to examine spontaneous brain activities (Sowndhararajan and Kim, 2016). Exposure to fragrance has been reported to affect brain activity and cognitive function (Iijima et al., 2009; Lin et al., 2022). The EEG power spectrum bands often employed in the investigation of brain activity include delta (0–4 Hz), theta (4–8 Hz), alpha (8–13 Hz), beta (13–30 Hz), and gamma (>30 Hz) waves (Sowndhararajan and Kim, 2016). EEG is a non-invasive, uncomplicated technology that assesses the olfactory system objectively (Krbot Skorić et al., 2015). Hence, in this study, EEG is used to evaluate subjects’ brain activity during the inhalation of *Cinnamomum camphora*.

Many types of aroma EOs are used to reduce anxiety and regulate mood (Masuo et al., 2021). A previous study found that inhaling lavender oil led to an increase in alpha power in EEG analysis, indicating better relaxation and an improved mood (Diego et al., 1998). Moreover, a study among women found that olfactory stimulation by Japanese cedar (*Cryptomeria japonica*) EO regulates mood states and suppresses sympathetic nervous activity, which improves mental health (Matsubara and Ohira, 2018). Recent scientific investigations have revealed that EOs obtained from various plant species, including the Korean fir, Siberian fir tree, *Inula helenium*, *Angelica gigas*, and peppermint, significantly affect brain wave activities (Diego et al., 1998; Matsubara et al., 2011; Sayorwan et al., 2012; Seo et al., 2016; Sowndhararajan et al., 2016, 2017; Kim et al., 2020; Lin et al., 2022).

In recent years, essential oils are widely used in several delivery populations, including children (Tripathy et al., 2023), adults (Lee et al., 2017), patients (Johnson et al., 2016; Lee and Hur, 2022), women (Matsumoto et al., 2013; Hong et al., 2018), teachers (Liu et al., 2013), and students (Choi et al., 2022). Given that college students are a unique population particularly prone to experiencing high levels of stress, anxiety, depression, and other psychological problems (Granieri et al., 2021; Faisal et al., 2022), aromatherapy has garnered significant research interest concerning the student population. Wakui et al. (2023) conducted a clinical trial involving 48 Japanese university students to assess the effects of bergamot essential oil. The findings indicated that the bergamot essential oil can alleviate psychological stress, improve sleep quality, and enhance morning wakefulness in the short term. Similarly, Özer et al. (2022) evaluated the efficacy of lemon essential oil in reducing test anxiety among nursing students. Their study demonstrated that lemon essential oil effectively reduced test anxiety by 43.3%. Furthermore, Kim and Song (2022) investigated the physiological and psychological relaxing effects of fir essential oil among university students. They utilized heart rate variability (HRV) and heart rate (HR) as physiological indicators, and the Profile of Mood States (POMS) and State–Trait Anxiety Inventory (STAI) as psychological indicators. Participants inhaled fir essential oil for 3 min, which resulted in lower ln(LF/HF) ratios, improved positive mood (vigor), and decreased negative moods and anxiety levels.

*Cinnamomum camphora*, a member of the Lauraceae family, is a deciduous broad-leaf tree native to regions such as South China, East Asia, India, and South Korea (Lee et al., 2022). Since China’s urbanization began in 1978, the *Cinnamomum camphora*, a typical indigenous street and park species, has been widely cultivated across urban areas (Zhang and Song, 2003). It is particularly prevalent from coastal regions to inland areas, including Taiwan and Hainan Province. Renowned for its valuable timber and diverse ecosystem services, this species serves as a cornerstone of urban forestry. It contributes significantly to the economic prosperity and environmental sustainability of numerous cities (Xiang et al., 2020). In addition, camphor trees have been extensively used in traditional Chinese herbal medicine, as nutritional supplements, perfume, health care items, and incense (Zhou and Yan, 2016). *Cinnamomum camphora* essential oil (CCEO) refers to a collection of volatile oil-like chemicals found in *C. camphora* that possess a distinct odor. It is utilized mainly in food, chemical, health care, and other industries (du et al., 2022). Furthermore, it holds significant value as a primary source of fragrances (Lee et al., 2022). CCEO comprises terpenes and phenylpropenes (Pragadheesh et al., 2013; Shi et al., 2013; Guo et al., 2016). These compounds possess several antioxidant, antidiabetic, anti-inflammatory (Lee et al., 2006), antibacterial (Zhou et al., 2016), and analgesic qualities (Xiao et al., 2021).

Despite extensive research having been conducted on the EOs of traditional medicinal plants and some tree species (Matsubara et al., 2011; Ikei et al., 2015; Han et al., 2017; Matsubara et al., 2017; Kim et al., 2018; Kim and Song, 2022; Zhang et al., 2022), studies on other types of plants are still minimal. Additionally, and the research indicators used have not been comprehensive. Investigations of the effects of *Cinnamomum camphora* essential oil on brain wave activity, the autonomic activity, and mood responses have rarely conducted. Therefore, this study aims to investigate the potential effects of inhaling CCEO on students’ physiological and psychological activities. The objective is to comprehensively evaluate its suitability for use in aromatherapy and to develop commercial values and resource utilization strategies for *Cinnamomum camphora*.

## 2 Materials and methods

### 2.1 Participants

A total of 43 healthy university students (26 females and 17 males, aged 18–25 years) at Fujian Agriculture and Forestry University were enrolled. None of the subjects had mental health problems, asthma, nasal diseases, or allergies to EOs. The objectives and schedule of the studies were clarified in advance, and participants were required to provide written informed consent before participation. Prior to the experiment, they were told to avoid excessive exercise, drinks containing alcohol, caffeine, smoking, or using any form of drug within 24 h of the investigation. This study was approved by the Human Research Ethics Committee of Fujian Provincial Hospital (K2019-03-006).

### 2.2 Olfactory stimulation

**Figure 1:**
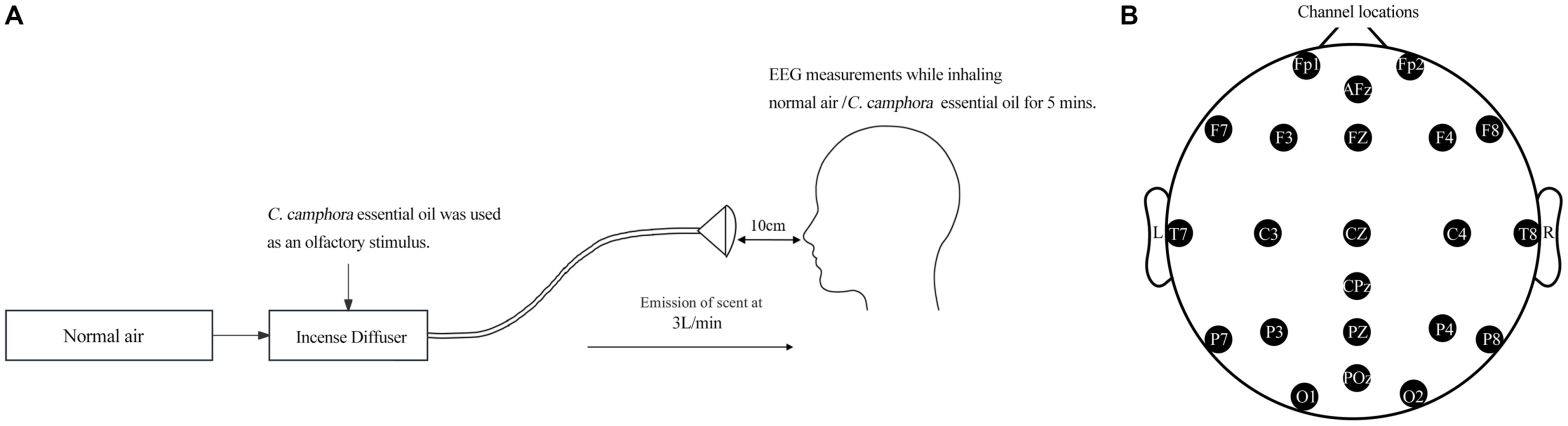
Experiment: **(A)** An incense diffuser; **(B)** EEG electrode placement locations using the international 10–20 system.

The CCEO was provided by the Department of Wood-Based Materials and Design, National Chiayi University. The essential oil was extracted from the leaves of *Cinnamomu camphora* using the steam distillation extraction method. For sensory evaluation, 1% of dilute CCEO was prepared using a colorless and odorless dipropylene glycol (DPG) solvent. This study employed a specialized incense diffuser capable of atomizing EOs and regulating the release duration. Specifically, the incense diffuser atomized the 1% CCEO solution, which was then further diluted with air before being provided to the subjects. CCEO was used as the olfactory stimulus, with normal room air as the control. The odor was administered for 5 min. Odors were presented to each subject by means of a funnel-shaped supplier fixed on the table, situated approximately 10 cm under the nose (Figure 1A).

A consistent temperature of 26°C and a humidity level of 50% was maintained in the experimental chamber (4 × 2.5 × 2.8 m). Participants were instructed to remain seated, close their eyes, and breathe regularly during the assessment. The EEG data were obtained 5 min before and throughout the five-minute inhaling process. The participants were not provided any prior information on the scent used in the studies.

### 2.3 GC–MS analysis of *Cinnamomum camphora* essential oil

The volatile aromatic components in the essential oil of *C. camphora* were identified by gas chromatography and mass spectrometry (GC–MS) analysis. GC–MS analysis was carried out with a Agilent 8,890-7000E equipped with a DB-WAX column (60 m × 320 μm × 0.25 μm). The GC oven temperature was kept at 50°C for 2 min, then heated to 250°C at a rate of 3°C/min, and maintained for 14 min. One μL of the sample was injected, and helium was used as a carrier gas at the rate of 1 mL/min. The injector temperature was set at 220°C and the ion source temperature was set at 230°C. The components of the oil were identified by the mass spectral library (NIST 20).

### 2.4 Experimental design

This study employed a single-group pretest–posttest research design to investigate the effects of inhalation of CCEO. The participants were provided with the goal and procedure of the study before the experiment. During the EEG measurement, they were instructed to maintain a state of stillness without any movement.

**Figure 2:**
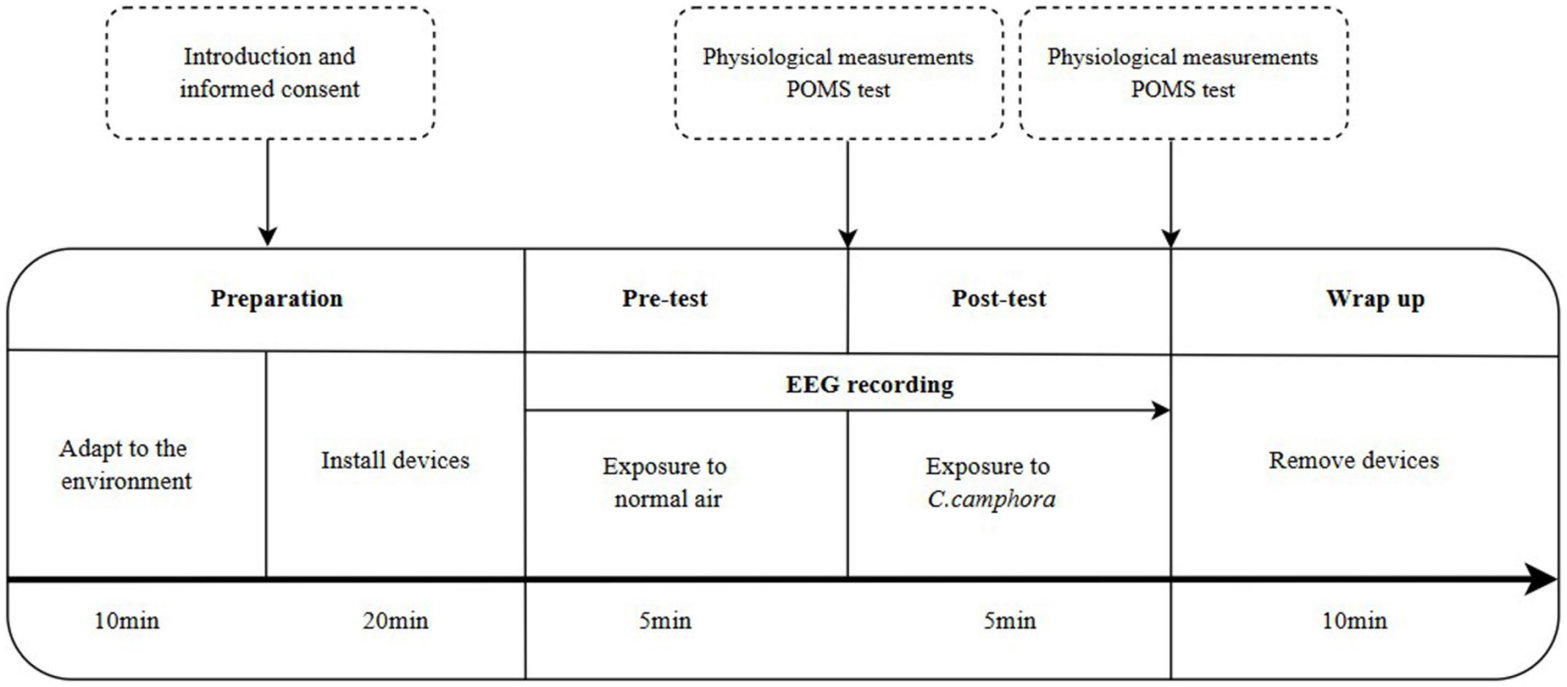
Experimental procedure.

The experimental procedure (Figure 2) was conducted in the following sequence: (1) the participants were initially given 10 min to adapt to the environment and provide their informed consent, after which the EEG cap was placed on their heads (20 min). (2) Each participant’s EEG was recorded for 5 min with eyes closed and while breathing normally; then, the baseline physiological parameters were measured, which included heart rate (HR), blood pressure (BP), pulse rate, blood oxygen saturation (SpO_2_). A POMS questionnaire was also administered and collected. (3) The CCEO was dispersed into the chamber using an incense diffuser without informing the participant for 5 min. The relevant physiological parameters and the POMS evaluation of the participants were once again measured. (4) After this, the EEG cap was removed. The entire experimental process lasted approximately 50 min.

### 2.5 Data analysis

The average power values [microvolt (μv^2^/Hz)] were computed for a total of 25 EEG analysis indicators (Table 1). The topographical mapping (t-mapping) of EEG wave was constructed by using the EEGLAB12.0 software program. T-maps can clearly illustrate the differences in EEG power spectra. Data were shown as mean ± SEM. The measurements were taken before and after the CCEO inhalation. All of the data were analyzed using SPSS version 26.0, and the results’ significance was determined using paired *t*-tests (*p* < 0.05) to examine trends.

**Table 1:** The abbreviations, full names, and wavelength ranges of the EEG power spectrum indices.

No.	Indices	EEG power spectrum indicators	EEG power spectrum indicators (Hz)
1	AT	Absolute theta power spectrum	4 ∼ 8
2	AA	Absolute alpha power spectrum	8 ∼ 13
3	AB	Absolute beta power spectrum	13 ∼ 30
4	AG	Absolute gamma power spectrum	30 ∼ 50
5	ASA	Absolute slow alpha power spectrum	8 ∼ 11
6	AFA	Absolute fast alpha power spectrum	11 ∼ 13
7	ALB	Absolute low beta power spectrum	12 ∼ 15
8	AMB	Absolute mid beta power spectrum	15 ∼ 20
9	AHB	Absolute high beta power spectrum	20 ∼ 30
10	RT	Relative theta power spectrum	(4 ∼ 8)/(4 ∼ 50)
11	RA	Relative alpha power spectrum	(8 ∼ 13)/(4 ∼ 50)
12	RB	Relative beta power spectrum	(13 ∼ 30)/(4 ∼ 50)
13	RG	Relative gamma power spectrum	(30 ∼ 50)/(4 ∼ 50)
14	RSA	Relative slow alpha power spectrum	(8 ∼ 11)/(4 ∼ 50)
15	RFA	Relative fast alpha power spectrum	(11 ∼ 13)/(4 ∼ 50)
16	RLB	Relative low beta power spectrum	(12 ∼ 15)/(4 ∼ 50)
17	RMB	Relative mid beta power spectrum	(15 ∼ 20)/(4 ∼ 50)
18	RHB	Relative high beta power spectrum	(20 ∼ 30)/(4 ∼ 50)
19	RST	Ratio of SMR to theta	(12 ∼ 15)/(4 ∼ 8)
20	RMT	Ratio of mid beta to theta	(15 ∼ 20)/(4 ∼ 8)
21	RSMT	Ratio of (SMR ~ mid beta) to theta	(12 ∼ 20)/(4 ∼ 8)
22	RAHB	Ratio of alpha to high beta	(8 ∼ 13)/(20 ∼ 30)
23	SEF50	Spectral edge frequency 50%	4 ∼ 50
24	SEF90	Spectral edge frequency 90%	4 ∼ 50
25	ASEF	Spectral edge frequency 50% of alpha spectrum band	8 ∼ 13

### 2.6 Physiological measurements

#### 2.6.1 EEG recording

According to the International 10–20 System, the EEG recordings were made using a SMARTING 24-channels electrode cap. The electrodes were positioned on the scalp at Fp1, Fp2, F3, F4, C3, C4, P3, P4, O1, O2, M1, M2, F7, F8, T7, T8, P7, P8, Fz, Cz, Pz, AFz, CPz, POz (Figure 1B). The electrodes were referenced to the ipsilateral earlobe electrodes, and M1 and M2 channels were used as reference electrodes. The EEG data was consistently captured at a sample frequency of 500 Hz, completing band-pass filtering within the frequency range of 0.5 to 50 Hz, followed by applying a 50 Hz notch filter. The EEG raw data was filtered and removed eye-blink or motor artifacts for each channel using MATLAB R2013b software. Power spectral data were analyzed using Fast-Fourier transform method (FFT). The EEG wave t-mapping was generated using the EEGLAB12.0 software program. The electrode gel (Wuhan Greentek Pty. Ltd., China) is applied into each electrode to connect with the surface of the scalp in order to drop the electric resistance of the scalp below 5 kΩ. The mean power values (μv^2^/Hz) are calculated, and the frequency bands such as alpha, beta, theta, gamma (25 indices) are recorded. EEG data will be compared between, before and during inhalation. The average power values were established to evaluate the effects of CCEO inhalation on the whole-brain area.

#### 2.6.2 HR, BP, pulse, and SpO_2_

A BP monitor (HEM-92200 L, Omron DALIAN, Co., Ltd., China) was used to measure HR, BP, and pulse. SpO_2_ was measured by a finger monitor (Philips DB18). Each participant’s BP and HR were recorded thrice, consecutively, and the mean value of these measurements was computed. Systolic blood pressure (SBP) and diastolic blood pressure (DBP) are the two fundamental constituents of BP. These values and HR were used to assess how stress affects the cardiovascular system (Rajoo et al., 2019), with higher values indicating greater stress.

#### 2.6.3 Psychological measurements

The Profile of Mood State (POMS) test, created by Grove and Prapavessis (1992) and simplified by Beili (1995) for Chinese people, was employed to measure the psychological impacts of olfactory stimulation. It is a self-reporting questionnaire consisting of 40 items designed to assess mood states across the following 7 dimensions: “tension,” “depression,” “anger,” “fatigue,” “panic,” “energy,” and “self-esteem.” High scores in the positive subscale (energy and self-esteem) and low scores in all the other subscales reflect a positive mood or feeling (Yoshihara et al., 2011). The total mood disturbance (TMD) score was calculated as follows: [(tension +depression + anger + fatigue + panic – energy – self-esteem) +100] (Lin et al., 2019). An improved emotional state indicates a lower TMD score (McNair et al., 1992). A higher TMD score indicates a more significant level of mood disturbance (Yoshihara et al., 2011).

## 3 Results

### 3.1 Chemicals in *Cinnamomum camphora*

The volatile aromatic components in the essential oil of *Cinnamomum camphora* were identified by gas chromatography and mass spectrometry (GC–MS) analysis. Nine major substances were identified with a search similarity of over 90% and a relative content exceeding 1%, accounting for approximately 94.88% of the total components. CCEO is mainly composed of monoterpenes hydrocarbons (Table 2). The primary constituents in CCEO are camphor (32.28%), followed by α-pinene (13.74%), p-cymene (13.63%), β-pinene (11.89%), γ-terpinene (9.29%), 1,8-cineole (5.31%), α-terpinene (3.79%), terpinen-4-ol (2.64%), and terpinolene (2.31%).

**Table 2:** The main chemical composition of the essential oil from the *Cinnamomum camphora*.

S. number	Component	Retention time (min)	Formula	Peak area (%)
1	α-Pinene	8.53	C₁₀H₁₆	13.74
2	β-Pinene	9.98	C₁₀H₁₆	11.89
3	α-Terpinene	11.43	C₁₀H₁₆	3.79
4	p-Cymene	11.72	C₁₀H_14_	13.63
5	1,8-Cineole	11.96	C_10_H_18_O	5.31
6	γ-Terpinene	12.97	C₁₀H₁₆	9.29
7	Terpinolene	14.03	C₁₀H₁₆	2.31
8	Camphor	16.07	C₁₀H₁₆O	32.28
9	Terpinen-4-ol	17.25	C₁₀H_18_O	2.64

### 3.2 Physiological responses

#### 3.2.1 Inhalation of CCEO on EEG power spectrum values

**Figure 3:**
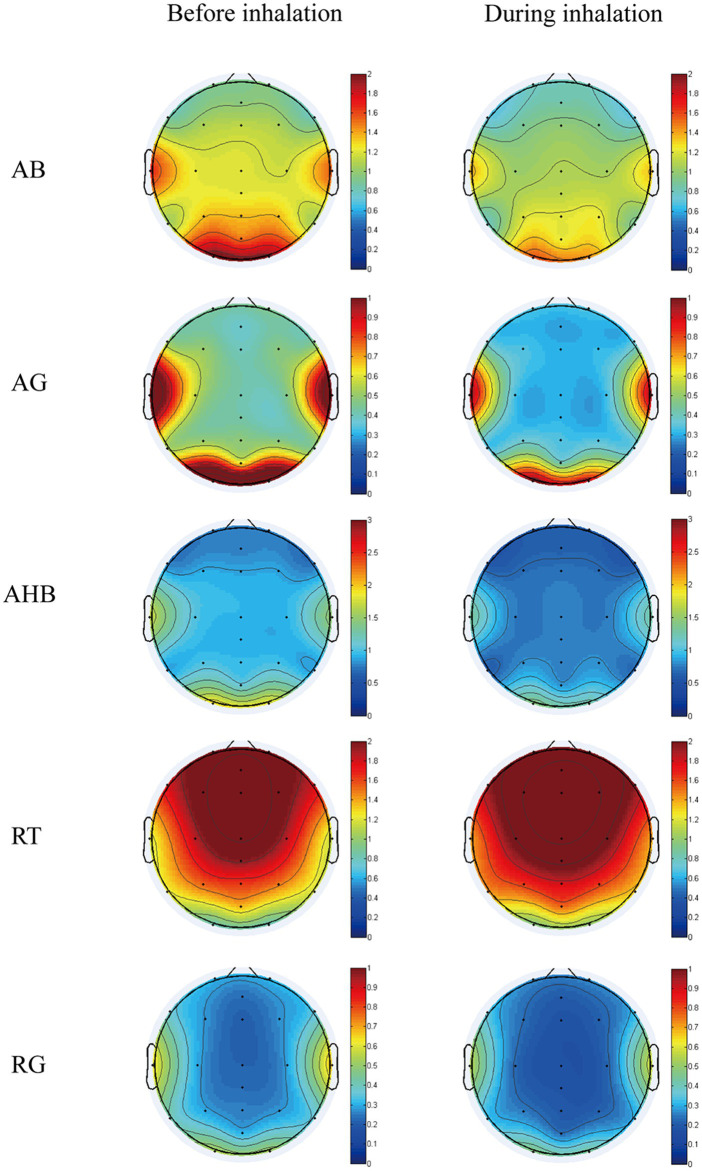
The t-mapping of EEG changes before and during the inhalation of CCEO. AB, absolute beta; AG, absolute gamma; AHB, absolute high beta; RT, relative theta; RG, relative gamma.

Table 3 depicts the significant changes in EEG power spectrum values before and during the CCEO inhalation. Among the 25 EEG indices analyzed, statistically significant differences were observed in 5. Figure 3 depicts the t-mapping of considerable EEG power spectrum value changes.

**Table 3:** Effect of inhalation of *C. camphora* on EEG power spectrum values.

Indice	Site	Before inhalation (μv^2^/Hz)	During inhalation (μv^2^/Hz)	*t* test	*p* value*
AB	P7	1.051 ± 0.549	0.313 ± 0.194	2.381	0.022*
AG	Fp1	0.577 ± 0.292	0.448 ± 0.344	2.206	0.033*
	Fp2	0.462 ± 0.326	0.316 ± 0.183	2.402	0.021*
	F3	0.501 ± 0.562	0.311 ± 0.176	2.1	0.042*
	F4	0.473 ± 0.470	0.298 ± 0.179	2.305	0.027*
	C3	0.566 ± 0.672	0.312 ± 0.175	2.392	0.022*
	C4	0.461 ± 0.326	0.305 ± 0.178	2.911	0.006**
	P3	0.501 ± 0.341	0.363 ± 0.259	2.065	0.046*
	P4	0.479 ± 0.321	0.330 ± 0.226	2.6	0.013*
	P7	0.544 ± 0.437	0.316 ± 0.197	3.13	0.003**
	P8	0.483 ± 0.337	0.346 ± 0.248	2.393	0.022*
	Fz	0.400 ± 0.283	0.289 ± 0.163	2.313	0.026*
	Cz	0.420 ± 0.288	0.297 ± 0.173	2.503	0.017*
	Pz	0.489 ± 0.327	0.327 ± 0.208	2.75	0.009**
	AFz	0.378 ± 0.274	0.271 ± 0.164	2.228	0.032*
	CPz	0.426 ± 0.291	0.294 ± 0.174	2.601	0.013*
	POz	0.627 ± 0.533	0.399 ± 0.359	2.236	0.031*
AHB	Fp1	0.771 ± 0.447	0.599 ± 0.291	2.237	0.031*
	Fp2	0.794 ± 0.430	0.600 ± 0.260	2.508	0.016*
	F3	0.870 ± 0.594	0.668 ± 0.296	2.274	0.029*
	F4	0.875 ± 0.529	0.673 ± 0.317	2.206	0.033*
	C3	1.009 ± 0.731	0.728 ± 0.346	2.321	0.026*
	C4	0.925 ± 0.469	0.734 ± 0.350	2.267	0.029*
	P3	0.998 ± 0.512	0.802 ± 0.384	2.065	0.046*
	F7	0.771 ± 0.567	0.564 ± 0.315	2.145	0.038*
	T7	1.708 ± 2.017	1.212 ± 1.401	1.244	0.221
	P7	0.891 ± 0.595	0.605 ± 0.303	2.57	0.014*
	P8	0.857 ± 0.495	0.674 ± 0.424	2.098	0.042*
	Fz	0.876 ± 0.446	0.710 ± 0.325	2.132	0.039*
	Cz	0.932 ± 0.466	0.756 ± 0.368	2.111	0.041*
	AFz	0.766 ± 0.416	0.618 ± 0.297	2.065	0.046*
	POz	1.084 ± 0.625	0.823 ± 0.446	2.25	0.03*
RT	Fp1	0.771 ± 0.447	2.115 ± 0.917	−9.01	0.000***
	Fp2	0.793 ± 0.430	2.167 ± 0.931	−8.672	0.000***
	F3	0.890 ± 0.594	2.317 ± 0.875	−8.733	0.000***
	F4	0.875 ± 0.528	2.327 ± 0.893	−9.106	0.000***
	C3	1.009 ± 0.730	2.081 ± 0.827	−6.049	0.000***
	C4	0.924 ± 0.468	2.058 ± 0.836	−7.595	0.000***
	P3	0.997 ± 0.511	1.659 ± 0.747	−4.863	0.000***
	P4	0.985 ± 0.511	1.658 ± 0.716	−5.415	0.000***
	O1	1.912 ± 1.594	0.942 ± 0.518	3.731	0.001**
	O2	2.065 ± 0.842	0.990 ± 0.564	6.175	0.000***
	Fz	1.495 ± 0.577	2.529 ± 0.577	−5.268	0.000***
	Cz	0.817 ± 0.398	2.373 ± 0.871	−9.621	0.000***
	Pz	0.811 ± 0.448	1.818 ± 0.793	−6.287	0.000***
	AFz	1.779 ± 0.761	2.392 ± 0.924	−2.781	0.008**
RG	C3	0.295 ± 0.202	0.219 ± 0.147	2.049	0.047*
	C4	0.288 ± 0.181	0.213 ± 0.144	2.208	0.033*

AB, high beta; AG, absolute gamma; AHB, absolute high beta; RT, relative theta; RG, relative gamma. Values represent the mean ± SEM. *Significant difference (*p < 0.05, **p < 0.01, ***p < 0.001).

During exposure to CCEO, a decrease in AB (13–30 Hz) was observed in the left temporal region (P7). In addition, a notable reduction in absolute gamma (30–50 Hz) occurred in frontal (Fp1, Fp2, F3, F4, C3, C4, Fz, AFz), central (Cz, CPz), and parietal (P3, P4, P7, P8, Pz, POz) regions, while relative gamma decreased in the central region (C3, C4). A significant reduction of absolute high beta (20-30 Hz) was also observed in all the regions (Fp1, Fp2, F3, F4, F7, Fz, AFz, C3, C4, CZ, P3, POz, T7, P7, P8). It was also noteworthy that except for the obvious decreasing trend in the occipital region (O1, O2), a notable increase was identified in relative gamma waves over other regions (Fp1, Fp2, F3, F4, C3, C4, P3, P4, Fz, CZ, Pz).

#### 3.2.2 Impact of the inhalation of CCEO on brain regions

We separated the recording electrodes into five sections based on the corresponding cortex beneath the skull to study the brain activities of each specified region: the frontal, temporal, central, parietal, and occipital areas (Liu et al., 2013). Single-channel analysis revealed that the CCEO could induce notable changes in 5 out of 25 EEG indices. We established distinct electrode groups dedicated to specific brain regions to investigate the activities associated with each of them. The frontal area comprises the following electrode placements: Fp1, Fp2, F3, F4, F7, F8, FZ, and AFz; T7 and T8 make up the temporal area of the brain; the central region comprises C3, C4, CZ, and CPz; the parietal area comprises P3, P4, P7, P8, and POz; and O1, and O2 make up the occipital area.

**Figure 4:**
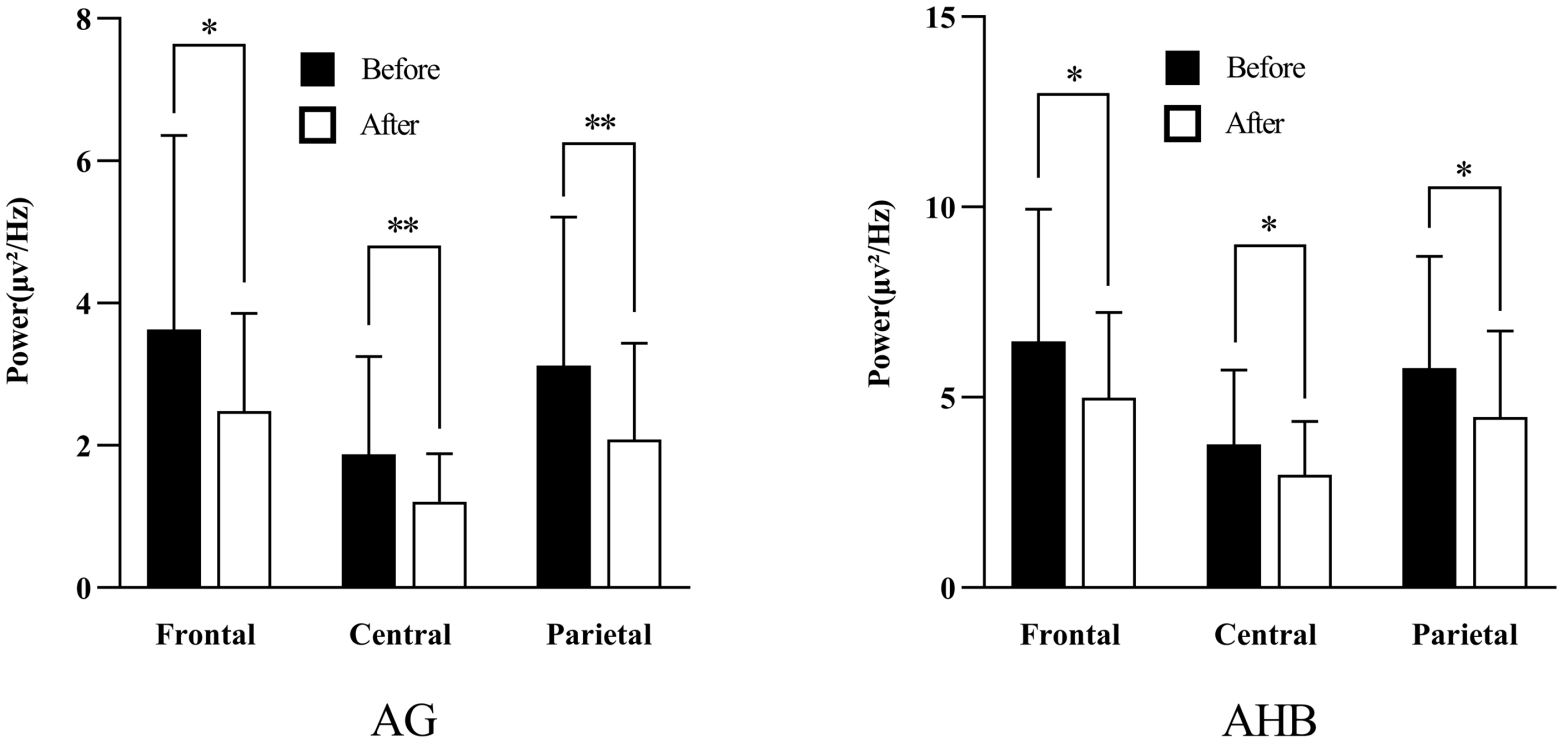
Representative brain wave power after inhaling *C. camphora* essential oil. AG, absolute gamma; AHB, absolute high beta. *Significant difference (*p < 0.05, **p < 0.01, ***p < 0.001).

Following the CCEO inhalation, as shown in Figure 4, there was a significant decrease in absolute gamma (AG, 30–50 Hz) in frontal (3.62 to 2.48 μv^2^/ Hz), central (1.87 to 1.20 μv^2^/ Hz), and parietal (3.12 to 2.08 μv^2^/Hz) regions. Simultaneously, significant changes in absolute high beta (AHB, 20-30 Hz) wave power were observed in frontal (6.46 to 4.99 μv^2^/ Hz), central (3.77 to 2.96 μv^2^/ Hz), and parietal (5.76 to 4.48 μv^2^/Hz) regions. The overall results indicate that CCEO inhalation significantly affects the frontal, central, and parietal areas.

#### 3.2.3 Effect of CCEO inhalation on HR, BP, pulse, and SpO_2_

CCEO inhalation had no statistically significant impact on participants’ HR, SBP, or SpO_2_ (Table 4). Nevertheless, it seemed to considerably affect the DBP and pulse rate. After inhaling CCEO for 5 min, participants’ DBP significantly decreased from 68.19 mmHg to 65.95 mmHg (*p* < 0.05), although the SBP remained unaffected. On the other hand, the pulse exhibited a significantly decreasing trend compared to the baseline session (*p* < 0.05), from 76.63 bpm to 72.02 bpm.

**Table 4:** Changes in HR, BP, pulse, and SpO_2_ after the CCEO intervention.

Parameters	Before inhalation	During inhalation	*t* test	*p* value*
HR (beats/min)	74.25 ± 10.81	72.15 ± 14.23	1.292	0.204
SBP (mm Hg)	103.98 ± 12.11	102.57 ± 12.68	1.663	0.103
DBP (mm Hg)	68.19 ± 9.35	65.95 ± 8.29	2.497	0.016*
Pulse (bpm)	76.63 ± 12.02	72.02 ± 9.91	4.265	0.000***
SpO_2_ (%)	97.34 ± 0.90	97.48 ± 1.03	−1.000	0.322

Heart rate (HR); Systolic blood pressure (SBP); Diastolic blood pressure (DBP); Blood oxygen saturation (SpO_2_). Values represent the mean ± SEM. *Significant difference (*p < 0.05, ***p < 0.001).

### 3.3 Psychological responses

**Figure 5:**
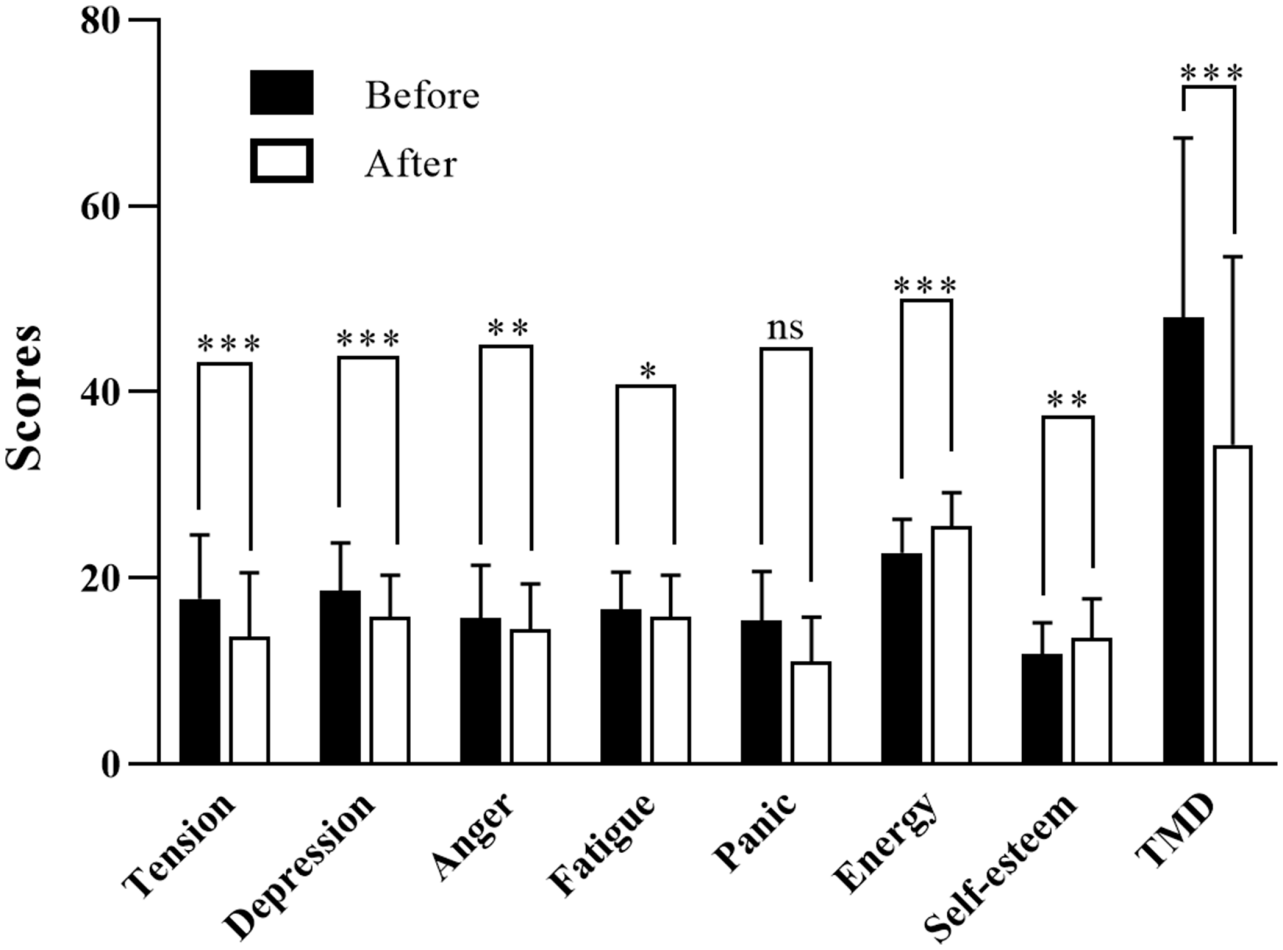
Changes in POMS scores after the CCEO intervention. TMD, Total Mood Disturbance, *N* = 43, values represent the mean ± SEM. *Significant difference (ns > 0.05, *p < 0.05, **p < 0.01, ***p < 0.001).

The findings from POMS revealed statistically significant impacts on the subjects, indicating that the CCEO inhalation could reduce anxiety levels and improve mood. As shown in Figure 5, the changes considerably decreased the mean scores of tension from 17.77 to 13.72 (*p* < 0.001), depression from 18.65 to 15.85 (*p* < 0.001), anger from 15.72 to 14.42 (*p* < 0.05), and confusion from 15.45 to 10.95 (*p* < 0.001). The average score for these negative mood subscales demonstrates a statistically significant decline following exposure to the aroma of CCEO. Besides, energy and self-esteem (passive indicator) scores improved greatly, with energy from 22.67 to 25.55 (*p* < 0.001), and self-esteem from 11.77 to 13.47 (*p* < 0.01), following the inhalation of CCEO. Further, the TMD scores after stimulation were significantly lower than baseline (*p* < 0.01). The POMS thus revealed many positive findings indicating that CCEO inhalation significantly enhances mood and increases positive emotion.

## 4 Discussion

Numerous authors have previously reported the influence of the inhalation of EOs (lavender, rose, and agarwood oils), as well as aromatic components, on the psychological and physiological states of humans (Masago et al., 2000; Kober et al., 2013; Angelucci et al., 2014; Sowndhararajan and Kim, 2016). This current experiment revealed several possible benefits associated with exposure to *C. camphora*, eliciting multiple physiological and psychological responses, based on EEG and autonomous system assessments.

Regarding the chemical composition, the primary constituents of the CCEO are camphor (32.68%), α-pinene (13.74%), p-cymene (13.63%), and β-pinene (11.89%), which may be responsible for its aromatic properties and potential impacts on humans. It is widely recognized that EOs or aromatic compounds create a positive atmosphere (Seo et al., 2016). Many studies have proved that inhalation of α-pinene and p-cymene can produce a specific anxiolytic effect (Balahbib et al., 2021; Donelli et al., 2023). A survey conducted by Harumi Ike (Ikei et al., 2016) revealed that olfactory stimulation using α-pinene resulted in a considerable reduction in heart rate, induced physiological relaxation, and increased parasympathetic nervous activity. Another study by Kim et al. (2018) demonstrated the positive effects of α-pinene and β-pinene on increasing the brain’s relaxation and alertness states. Zhou et al. (2021) reported that volatile organic compounds released from *Cinnamomum camphora* forests have therapeutic properties for older people. Although numerous variables, including production methods, location, plant age, harvest time, and meteorological conditions may impact variations in EO yield and composition (Dhouioui et al., 2016).

Physiologically, the alterations in BP and pulse are associated with stress (Zhang et al., 2022). Heart rate and blood pressure are commonly utilized to measure the psychophysiological effects of fragrances. An increase in these physiological activities may indicate anxiety, stress, or a stimulating effect, whereas a decrease generally signifies relaxation (Hongratanaworakit, 2004; Watanuki and Kim, 2005; Angelucci et al., 2014). According to earlier research, a pleasant smell or inhalation of EOs such as lavender, ylang-ylang, and bitter orange may induce a decrease in HR, indicating a relaxing effect (Bensafi, 2002; Zhang and Yao, 2019). Notably, the inhalation of essential oils or aromatic plant volatile oils can directly stimulate the olfactory system, prompting the brain to produce neurotransmitters (Watanuki and Kim, 2005). Consequently, this activation stimulates the autonomic nervous system, which modulates physiological functions such as heart rate, blood pressure, pulse, breathing, memory, and stress response (Sayorwan et al., 2012; Kim et al., 2018). After the CCEO intervention, a significant decrease was observed in DBP and the pulse rate showed a downward trend. These findings suggest that the inhalation of CCEO could beneficially alleviate stress levels.

With regard to the impact of aroma on EEG activity, a significant reduction in AB (13–30 Hz) and AHB (20–30 Hz) were observed during inhalation of CCEO. High beta wave activity is mostly associated with the brain’s high awareness/alertness state (Kim et al., 2018). A study reported that the inhalation of peppermint fragrance may stimulate the brain to remain active (Lin et al., 2022). Similarly, a reduction in beta wave activities is mostly linked to slower brain function (Lee et al., 2014; Kim et al., 2018). Consequently, this study indicates that the inhalation of CCEO leads to a reduction in beta activity, which in turn may decrease the level of alertness.

In this study, RT waves significantly increased in 12 out of 24 electrodes due to the inhalation of CCEO. It has been suggested that theta wave activity may help maintain attention when engaging in challenging tasks (Razumnikova, 2007; Greenberg et al., 2015). In addition, the process of creating memories is greatly influenced by theta waves (Sowndhararajan and Kim, 2016). Matsubara et al. (2011) reported that inhalation of the EO of *A. sibirica* during a visual display terminal task increased theta wave activities, indicating that the EO of *A. sibirica* may help in recovery from mental fatigue and prevent mental health disturbances. Our finding is in agreement with previous studies. CCEO may, therefore, have the potential to maintain attention, improve memory, and prevent certain mental health problems.

In addition, AG and RG waves also decreased during exposure to CCEO. Gamma waves are thought to enhance feature binding in sensory processing since they are observed during working-memory matching and several early sensory responses (Skinner et al., 2000; Wang et al., 2013). RG was utilized as a stress evaluation marker in the prefrontal brain (Minguillon et al., 2016). Yerin et al. (2021) reported that mixed EOs with sweet orange, lavender, and amyris could relax the brain and confirm a state of mental immersion through an increase in RG. This finding is similar to the effects of CCEO on human brain activity. Hence, CCEO exposure may help relieve stress and improve positive emotions.

In summary, CCEO seems to positively impact brain activities, leading to improved attention and cognitive abilities. Regarding brain regions, the findings suggest that CCEO inhalation significantly impacts the frontal, central, and parietal areas to a greater extent compared to other regions, which are in charge of movement, attention, and cognition/memory functions, respectively (Sowndhararajan and Kim, 2016). Thus, experiencing a floral scent such as CCEO can potentially foster brain functions such as memory, attention, and cognition and evoke a physiological effect similar to relaxation.

Psychologically, CCEO may be beneficial to enhance the emotional state of individuals (Morfeld et al., 2007). This helps explain the shared experience of EO benefiting mood and emotions. This study also supports previous research on the benefits of CCEO in terms of improving mood (Du et al., 2022; Lee et al., 2022; Ueda et al., 2023). Ueda et al. (2023) evaluate the effects of inhaling CCEO on human brain and memory function using EEG and a working memory task. The study revealed that participants exhibited a preference for CCEO, and an enhancement in positive mood. These findings may be attributed to the specific concentration of CCEO and the duration of inhalation time. The POMS has been used to assess the effects of EO, such as yuzu (Matsumoto et al., 2016), lavender (Matsumoto et al., 2013), Japanese plum blossom (Jo et al., 2013), and white jasmine (Xiong et al., 2023), which have been proven to reduce the POMS score. In the present study, the POMS scores revealed a significant decrease in four negative themes and TMD values after inhaling CCPO, while the positive theme scores (energy, self-esteem) exhibited a significant increase. These findings suggest that, compared to odorless treatment, aromatherapy using CCPO may not only alleviate negative emotions such as anxiety, anger, and depression but also promote positive emotions.

However, this study has several potential limitations. Firstly, our research employs a brief duration of stimulation of *Cinnamomum camphora* essential oil. In subsequent times, obtaining extensive data spanning several days, months, and years will be vital. Secondly, the EEG recordings were adjusted to account for subjects’ eye movements, which may introduce some deviations. Thirdly, it was conducted with university students in their 20s. To enhance the generalizability of the findings, further studies should be predicated upon more expansive sample size, including diverse age ranges and demographic groupings.

## 5 Conclusion

In brief, the CCEO appears to have physiologically and psychologically positive stimulatory effects. These significant findings regarding the impact of inhaling CCEO on university students can be summarized as follows: (1) reduced sympathetic nervous activity; (2) improved energy and depressed negative feelings, such as tension and depression; (3) decreased alertness, improved attention, and memory, primarily affecting the brain’s frontal, central, and parietal areas. Thus, short-term inhalation of CCEO can cause physiological and psychological relaxation in university students. This study offers a valuable insight into the new value and function of *Cinnamomum camphora* essential oil, as it supports students’ well-being and can be used in aromatherapy for positive psychophysiological conditions, thereby fostering the development of complementary approaches and ecosystem service value of *Cinnamomum camphora*, especially in natural education, natural therapy and ecotourism.

## Data availability statement

The datasets presented in the study are included in the article/supplementary material, further inquiries can be directed to the corresponding author.

## Ethics statement

The studies involving humans were approved by Ethics Committee of Fujian Provincial Hospital (K2019-03-006), China. The studies were conducted in accordance with the local legislation and institutional requirements. The participants provided their written informed consent to participate in this study.

## Author contributions

XG: Conceptualization, Data curation, Formal analysis, Investigation, Methodology, Project administration, Resources, Software, Supervision, Validation, Visualization, Writing – original draft, Writing – review & editing. YY: Data curation, Investigation, Methodology, Project administration, Software, Validation, Visualization, Writing – original draft, Writing – review & editing. TX: Conceptualization, Data curation, Project administration, Visualization, Writing – original draft. DY: Conceptualization, Software, Supervision, Visualization, Writing – original draft. SL: Project administration, Visualization, Writing – original draft. WC: Conceptualization, Funding acquisition, Supervision, Writing – review & editing.
